# Secondary Metabolite Profiling of Species of the Genus *Usnea* by UHPLC-ESI-OT-MS-MS

**DOI:** 10.3390/molecules23010054

**Published:** 2017-12-27

**Authors:** Francisco Salgado, Laura Albornoz, Carmen Cortéz, Elena Stashenko, Kelly Urrea-Vallejo, Edgar Nagles, Cesar Galicia-Virviescas, Alberto Cornejo, Alejandro Ardiles, Mario Simirgiotis, Olimpo García-Beltrán, Carlos Areche

**Affiliations:** 1 Departamento de Química, Facultad de Ciencias, Universidad de Chile, Las Palmeras 3425, Nuñoa, Santiago 7800024, Chile; fsalgado@ug.uchile.cl (F.S.); lauraalbornozh@gmail.com (L.A.); carmenc1012@gmail.com (C.C.); edgar.nagles@unibague.edu.co (E.N.); cesar.galicia@unibague.edu.co (C.G.-V.); 2 Research Center of Excellence CENIVAM, CIBIMOL, Universidad Industrial de Santander, Building 45, UIS, Carrera 27, Calle 9, Bucaramanga 680002, Colombia; elena@tucan.uis.edu.co; 3 Facultad de Ciencias Naturales y Matemáticas, Universidad de Ibagué, Carrera 22 Calle 67, Ibagué 730001, Colombia; c120121022@estudiantesunibague.edu.co; 4 Escuela de Tecnología Médica, Facultad de Medicina, Universidad Andrés Bello, Sazié 2315, Santiago 8370092, Chile; alberto.cornejo@unab.cl; 5 Facultad de Ciencias de la Salud, Universidad Arturo Prat, Casilla 121, Iquique 1100000, Chile; alardiles@unap.cl; 6 Instituto de Farmacia, Facultad de Ciencias, Universidad Austral de Chile, Campus Isla Teja, Valdivia 5090000, Chile; mario.simirgiotis@uach.cl or mario.simirgiotis@gmail.com; 7 Center for Interdisciplinary Studies on the Nervous System, Universidad Austral de Chile, Campus Isla Teja, Valdivia 5090000, Chile

**Keywords:** lichen, *Usnea*, natural product, Orbitrap™, UHPLC-MS-MS

## Abstract

Lichens are symbiotic associations of fungi with microalgae and/or cyanobacteria, which are considered among the slowest growing organisms, with strong tolerance to adverse environmental conditions. There are about 400 genera and 1600 species of lichens and those belonging to the *Usnea* genus comprise about 360 of these species. *Usnea* lichens have been used since ancient times as dyes, cosmetics, preservatives, deodorants and folk medicines. The phytochemistry of the *Usnea* genus includes more than 60 compounds which belong to the following classes: depsides, depsidones, depsones, lactones, quinones, phenolics, polysaccharides, fatty acids and dibenzofurans. Due to scarce knowledge of metabolomic profiles of *Usnea* species (*U. barbata*, *U. antarctica*, *U. rubicunda* and *U. subfloridana*), a study based on UHPLC-ESI-OT-MS-MS was performed for a comprehensive characterization of their secondary metabolites. From the methanolic extracts of these species a total of 73 metabolites were identified for the first time using this hyphenated technique, including 34 compounds in *U. barbata*, 21 in *U. antarctica*, 38 in *U. rubicunda* and 37 in *U. subfloridana*. Besides, a total of 13 metabolites were not identified and reported so far, and could be new according to our data analysis. This study showed that this hyphenated technique is rapid, effective and accurate for phytochemical identification of lichen metabolites and the data collected could be useful for chemotaxonomic studies.

## 1. Introduction

Lichens are symbiotic associations of fungi with microalgae and/or cyanobacteria. Lichens are among the slowest growing organisms with strong tolerance to adverse environmental conditions ranging from plains to the highest mountains of tropical to Arctic regions under xeric to aquatic conditions. Lichens can grow on or within rocks, soil, trees, shrubs, trunks, on bricks, leather and wood. They have been used since ancient times as sources of color dyes, cosmetics, and as medicine for the treatment of bronchitis, asthma, leprosy, burning sensation, spleen enlargement, heart diseases, stomach disorders, liver pain, inflammation, and vomiting [1,2].

Lichens are sources of phenolic secondary metabolites of different types, including phenols (orcinol and β-orcinol), quinones (parietin and emodin), dibenzofurans (pannaric acid), depsidones (salazinic acid), depsones (picrolichenic acid), depsides (homosekikaic acid), γ-lactones (protolichesterinic acid), pulvinic acid derivatives (vulpinic acid), and xanthones (lichexanthone). In addition other constituents as cyclodepsipeptides, phenylalanine derivatives, halogenated compounds, brominated acetylenic fatty acids, and acetogenins, macrolactone glycosides have also been reported from lichens [3,4].

On the other hand, the genus *Usnea* contains more the 360 species, which are distributed in polar and tropical regions and their morphology is characterized by the presence of a cartilaginous central axis composed by dense fungal hyphae. Usnic acid is the most typical and abundant compound in the genus *Usnea*, which gives yellow color to thalli. Traditionally, the genus *Usnea* has been used to treat diarrhea, ulcers, urinary infections, tuberculosis, pneumonia, stomachache, antifungal, and cattle fungal diseases. The phytochemistry from *Usnea* species has revealed the presence of almost 60 compounds, distributed among depsidones, depsides, depsones, lactones, quinones, polyphenolics, polysaccharides, fatty acids, and dibenzofurans [2].

New techniques like ultra-high performance liquid chromatography-diode array detection (UHPLC-DAD) coupled to an electrospray ionization tandem mass spectrometer (ESI-MS-MS) have emerged for identification and elucidation of metabolites in complex extracts [5,6,7,8,9]. The Q-Exactive Focus is a newly released fast high-resolution mass spectrometer used to detect and quantify small organic compounds (up to 2000 amu). The hyphenated Q-exactive focus instrument is a high-resolution accurate mass (HRAM) instrument which combines UHPLC-DAD with an Orbitrap™, a quadrupole (Q) and a high-resolution collision cell (HCD), which allows high resolution MS fragments [5,6,7]. This hyphenated technique is a strong weapon in the field of chemical lichenology and some lichens, namely: *Ramalina siliquosa*, *Parmotrema grayana, Heterodermia obscurata, Ramalina terebrata*, *Everniopsis trulla* have been studied under this technique [6,7,9,10].

Continuing our research on lichens, we have analyzed the phytochemical profile of four *Usnea* species for the first time based on UHPLC-DAD coupled with high resolution electrospray ionization tandem mass spectrometry (ESI-MS-MS).

## 2. Results and Discussion

Four *Usnea* species were studied in order to determine their metabolomics profiles and chemical fingerprints: *U. barbata* from Longavi, Chile; *U. antarctica* from Antarctica, *U. rubicunda* and *U. subfloridana* from Colombia.

### 2.1. Metabolomics in Usnea barbata

**Figure 1:**
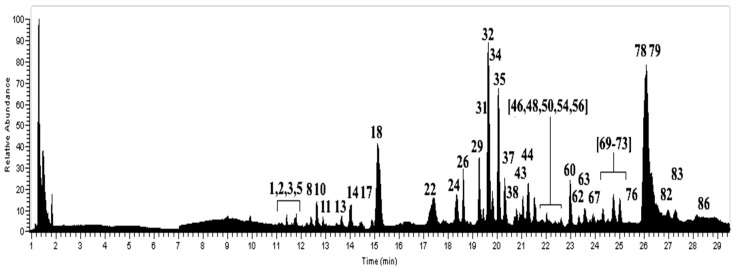
UHPLC-MS Chromatogram of *U. barbata*.

Forty-four peaks (Figure 1) were detected for the first time in a methanolic extract (Table S1 in Supplementary Information) using UHPLC/ESI/MS/MS in negative mode. Thirty-four compounds identified in this species were mainly depsides, depsidones, lipids, diphenylether derivatives and dibenzofurans.

#### ***Depsides*** 

Thirteen depsides were identified (peaks 8, 10, 22, 31, 48, 60, 63, 69–72, 79 and 86) using UHPLC with DAD and high-resolution mass spectrometry (HRMS) and MS^2^ analysis. Peak 8 was identified as barbatolic acid (molecular anion at *m*/*z* 389.0516) whose fragmentation produced a diagnostic MS^2^ ions at *m*/*z* 167.0342 and 121.0285. Peaks 10 and 22 were identified as thamnolic acid and haemathamnolic acid isomers, showing [M − H]^−^ ions at *m*/*z* 419.0619 and 403.0672, respectively. Peak 31 was identified as lecanoric acid, which showed an [M − H]^−^ ion at *m*/*z* 317.0668. Major diagnostic daughter MS ions of lecanoric acid were [M − H − C_8_H_6_O_3_]^−^, [M − H − C_8_H_8_O_4_]^−^ and [C_7_H_7_O_2_]^−^ (167.0343, 149.0237 and 123.0444 a.m.u., respectively). Peak 48 with a [M − H]^−^ pseudomolecular ion at *m*/*z* 467.0985 was identified as gyrophoric acid, which showed diagnostic daughter ions at *m*/*z* 317.0667, 167.0345, 149.0238 and 123.0443. Peak 60 was identified as diffractaic acid, whose molecular anion was at *m*/*z* 373.1294. Its fragmentation produced ions at *m*/*z* 297.1149 [M − H − C_2_H_4_O_3_]^−^, 181.0499 [M − H − C_11_H_12_O_3_]^−^, 177.0549 [M − H − C_10_H_12_O_4_]^−^, and 137.0600 [M − H − C_12_H_12_O_5_]^−^ confirming this depside. Peak 63 was identified as methyl-8-hydroxy-4-*O*-demethylbarbatate, which showed an [M − H]^−^ ion at *m*/*z* 375.1085. Peak 69 with a [M − H]^−^ ion at *m*/*z* 387.1452 was identified as divaricatic acid. The parent ion produced major diagnostic MS ions at *m*/*z* 195.0661 [M − H − C_11_H_12_O_3_]^−^, 177.0551 [M − H − C_11_H_14_O_4_]^−^ and 151.0759 [C_9_H_11_O_2_]^−^ confirming this depside. Peak 70 presented a pseudomolecular ion at *m*/*z* 359.1139, which produced fragmented ions at *m*/*z* 181.0503, 163.0394 and 137.0601, thus it was identified as barbatic acid. Peak 71 was identified as sekikaic acid (molecular anion at *m*/*z* 417.1556). The fragmentation of peak 71 produced diagnostic ions at *m*/*z* 225.0764 [M − H − C_11_H_12_O_3_]^−^, 209.0815 [M − H − C_11_H_12_O_4_]^−^, and 165.0915 [M − H − C_12_H_12_O_5_]^−^. Peak 72 were identified as 8-hydroxybarbatic acid, which showed an [M − H]^−^ ion at *m*/*z* 375.1088. Peak 79 and 86 were identified as atranorin and chloroatranorin, which showed [M − H]^−^ ions at *m*/*z* 373.0929 and 407.0541, respectively. The major diagnostic daughter ions were at *m*/*z* 177.0187 and 163.0394 a.m.u. for atranorin, while for chloroatranorin ions were at *m*/*z* 228.9906. These findings are in good agreement with the studies of Le Pogam et al. [8], Musharraf et al. [9] and Parrot et al. [10].

#### ***Depsidones*** 

Eight depsidones corresponding to peaks 18, 24, 29, 32, 35, 46, 50, and 73 were identified using UHPLC with DAD and HRMS-MS analysis. Peak 18 was identified as salazinic acid, which showed a [M − H]^−^ ion at *m*/*z* 387.0359. Its major diagnostic daughter ions were at *m*/*z* 269.0464, 241.0504, 151.0394 and 123.0445 a.m.u. Peak 24 was identified as connorstictic acid, which showed an [M − H]^−^ ion at *m*/*z* 373.0565. Peak 29 with a [M − H]^−^ pseudomolecular ion at *m*/*z* 401.0514 was identified as siphulellic acid, which showed diagnostic daughter ions at *m*/*z* 253.0505, 149.0238, and 123.0444. Galbanic acid was at the peak 32 (molecular anion at *m*/*z* 429.0463). The fragmentation of peak 32 produced ions at *m*/*z* 269.0456, 149.0237 and 123.0443. Peak 35 with a [M − H]^−^ ion at *m*/*z* 371.0409 was identified as norstictic acid. The parent ion produced major diagnostic MS ions at *m*/*z* 327.0509, 151.0393 and 123.0444 confirming this depsidone. Peak 46 and 50 were identified as α-acetylconstictic acid and stictic acid, which showed a [M − H]^−^ ion at *m*/*z* 443.0620 and 385.0567 respectively. The major diagnostic daughter ions were at *m*/*z* 383.0418, 343.0425 and 269.0457 a.m.u. for α-acetylconstictic, while for stictic acid ions were at *m*/*z* 357.0617, 313.0720 and 179.0347. Peak 73 was identified as lobaric acid (molecular anion at *m/z* 455.1712). The fragmentation of peak 38 also produced ions at *m*/*z* 411.1815 [M − H − CO_2_]^−^, 367.1909 [M − H − 2CO_2_]^−^, 352.1681 [M − H − 2CO_2_ − CH_3_]^−^, and 296.1048 [M − H − 2CO_2_ − C_5_H_11_]^−^ confirming this depsidone. These findings are in good agreement with the reports of Castro et al. [7], Le Pogam et al. [8] and Musharraf et al. [9].

#### ***Lipids*** 

Eleven polyhydroxylated lipids were identified (peaks 26, 34, 37, 38, 43, 54, 56, 67, 76, 82 and 83) using UHPLC-ESI-MS-MS analysis. Peak 26 with an [M − H]^−^ ion at *m*/*z* 375.2752 was identified as tetrahydroxyeicosanoic acid. Peak 34 showed an [M − H]^−^ ion at *m*/*z* 403.3067 was identified as tetrahydroxydocosanoic acid. Peak 37, 38 and 43 were identified as tetrahydroxytricosanoic acid (C_23_H_46_O_6_), tetrahydroxydocosanoic acid (C_22_H_44_O_6_) and 6-ethyl-6-*n*-pentylpentadecan-4,5,7,8,15-pentol-15-acetate (C_24_H_48_O_6_), which showed a [M − H]^−^ ion at *m*/*z* 417.3221, 403.3066 and 431.3379 respectively. Besides, peak 54, 56 and 67 were identified as tetrahydroxyhexacosanoic acid (C_26_H_52_O_6_), nonahydroxyoctacosanoic acid (C_28_H_56_O_11_) and heptahydroxytricosatrienoic acid (C_23_H_40_O_9_), which showed a [M − H]^−^ ion at *m*/*z* 459.3693, 567.3667 and 459.2602 respectively. Finally, peak 76, 82 and 83 presented an [M − H]^−^ ion at *m*/*z* 473.2756, 251.2016 and 295.1916, respectively. They were identified as the fatty acids tetrahydroxytrioxotetracosanoic acid (C_24_H_42_O_9_), hexadecadienoic acid (C_16_H_28_O_2_), and dihydroxyheptadecatrienoic acid (C_17_H_28_O_4_), respectively.

#### ***Diphenylethers*** 

A diphenylether (peak 62) was detected in the methanolic extract using UHPLC-DAD-MS-MS analysis. Peak 62 was identified as β-alectoronic acid, which showed a [M − H]^−^ ion at *m*/*z* 511.1976. Its major diagnostic daughter ions were at *m*/*z* 369.1339, 247.0969, and 163.0396 a.m.u.

#### ***Dibenzofurans*** 

Usnic acid with a [M − H]^−^ ion at *m/z* 343.0823 was evidenced as peak 78. The main daughter ions of peak 78 were [M − H − CH_3_]^−^, [M − H − C_4_H_3_O_2_]^−^ and [M − H − C_5_H_3_O_3_]^−^ (328.0591, 259.0609 and 231.0661 a.m.u., respectively).

#### ***Unknown Compounds*** 

Ten other compounds were detected (peaks 1–5, peaks 11, 13, 14, 17 and peak 44) but none was identified.

### 2.2. Metabolomics in Usnea antarctica

**Figure 2:**
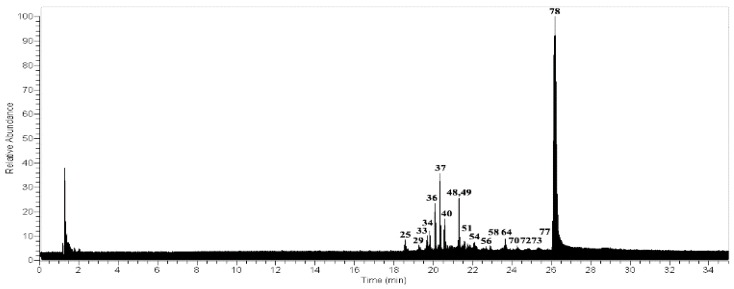
UHPLC-MS Chromatogram of *U. antarctica*.

Twenty-one peaks (Figure 2) were identified for the first time in the methanolic extract (Table 1) using UHPLC/ESI/MS/MS in negative mode. Among the classes of compounds identified we can cite depsides, depsidones, lipids, and dibenzofurans.

#### ***Depsides*** 

Five depsides were identified (peaks 48, 58, 63, 70 and 72) using UHPLC with DAD and HRMS and MS^2^ analysis. Peak 48 was identified as gyrophoric acid. Peak 58 and 72 were identified as baeomycesic acid and 8-hydroxybarbatic acid, showing [M − H]^−^ ions at *m*/*z* 373.0915 and 375.1070, respectively. Peak 63 and 70 were identified as methyl-8-hydroxy-4-*O*-demethylbarbatate and barbatic acid respectively. These findings are in good agreement with the studies of Castro et al. [7], Le Pogam et al. [8], Musharraf et al. [9] and Parrot et al. [10].

#### ***Depsidones*** 

Four depsidones corresponding to the peaks 24, 36, 40, and 73 were identified using UHPLC with DAD and HRMS-MS analysis. Peak 24 was identified as connorstictic acid. Peak 36 was identified as fumarprotocetraric acid (C_22_H_16_O_12_), which showed an [M − H]^−^ ion at *m*/*z* 471.0547. The fragmentation of this peak 36 produced ions at *m*/*z* 355.0441, 311.0545 and 115.0023. Peak 40 with a [M − H]^−^ pseudomolecular ion at *m*/*z* 387.0705 was identified as hypoconstictic acid, which showed diagnostic daughter ions at *m*/*z* 343.0808 and 299.0923. Finally, peak 73 was identified as lobaric acid. These findings are in good agreement with the reports of Le Pogam et al. [8], Musharraf et al. [9] and Parrot et al. [10].

#### ***Lipids*** 

Nine polyhydroxylated lipids were identified (peaks 30, 33, 34, 37, 43, 49, 51, 54 and 56) using UHPLC-ESI-MS-MS analysis [6,7].

#### ***Other Compounds*** 

Finally, an aromatic compound (peak 77) and two dibenzofurans (peak 64 and 78) corresponding to ethyl-4-*O*-methylolivetolcarboxylate, usnic acid and placodiolic acid were identified in this extract, respectively. Placodiolic acid showed a molecular anion at *m/z* 375.1069. The fragmentation of this compound produced ions at *m*/*z* 343.0806 [M − H − CH_3_OH]^−^, 259.0597 [M − H − C_5_H_8_O_3_]^−^, and 231.0648 [M − H − C_6_H_8_O_5_]^−^ confirming this dibenzofuran.

### 2.3. Metabolomics in Usnea rubicunda

**Figure 3:**
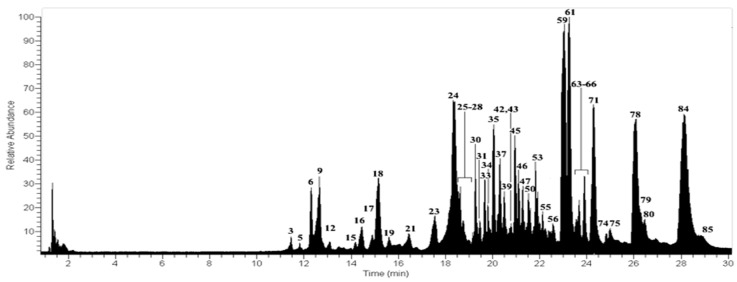
UHPLC-MS Chromatogram of *U. rubicunda*.

Forty-five peaks (Figure 3) were detected for the first time in the methanolic extract of *U. rubicunda* using UHPLC/ESI/MS/MS in negative mode (Table 1). Thirty-eight compounds were identified in *U. rubicunda* and among them are depsides, depsidones, lipids, diphenylether and dibenzofurans. These findings are in good agreement with the reports of Cornejo et al. [6], Castro et al. [7], Le Pogam et al. [8], Musharraf et al. [9] and Parrot et al. [10].

#### ***Depsides*** 

Nine depsides were identified and assigned to the peaks 6, 15, 19, 31, 53, 63, 71, 74 and 79 using UHPLC-DAD-MS-MS. Peak 6 and peak 15 were identified as conprotocetraric acid and squamatic acid (molecular anions at *m*/*z* 375.0724 and 389.0880, respectively). Peak 19 was identified as conhypoprotocetraric acid which showed an [M − H]^−^ ion at *m*/*z* 359.0775 and its fragmentation MS^2^ ion at *m*/*z* 253.0870 [M − H − C_2_H_2_O_5_]^−^. Peak 31, 53, 63, 71, 74 and 79 were identified as lecanoric acid, 4-*O*-methylnorsekikaic acid, methyl-8-hydroxy-4-*O*-demethylbarbatate, sekikaic acid, boninic acid and atranorin, respectively.

#### ***Depsidones*** 

Ten depsidones corresponding to peaks 9, 16, 18, 21, 23-25, 35, 39, and 50 were identified (Table 1). Peak 9 was identified as siphulellic acid isomer ([M − H]^−^ ion at *m*/*z* 401.0517), its fragmentation produced ions at *m*/*z* 359.0410 and 240.0427. Peak 16, 18, 21, 23-25, 35, 39 and 50 were identified as protocetraric acid, salazinic acid, constictic acid, hypoconstictic acid, connorstictic acid, menegazziaic acid, norstictic acid, cryptostictic acid, and stictic acid respectively.

#### ***Lipids*** 

Eighteen polyhydroxylated lipids were identified at the peaks 26, 28, 30, 33, 34, 37, 42, 43, 47, 55, 59, 61, 65, 66, 75, 80, and 84, 85 using UHPLC-ESI-MS-MS analysis.

#### ***Unknown Compounds*** 

Seven compounds were detected in this extract by the peaks 2, 3, 5, 12, 17, 27 and peak 45 but none of them were identified.

#### ***Other Compounds*** 

Finally, a dibenzofuran (peak 78) corresponding to usnic acid was detected and identified in this extract.

### 2.4. Metabolomics in Usnea subfloridana

**Figure 4:**
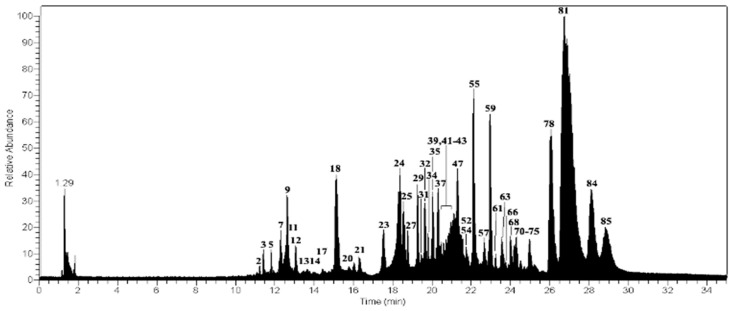
UHPLC-MS Chromatogram of *U. subfloridana*.

Forty-five (Figure 4) peaks were detected for the first time in the methanolic extract of *U. subfloridana* using UHPLC/ESI/MS/MS in negative mode (Table 1). Thirty-seven compounds were identified in this species which can be arranged as depsides, depsidones, lipids and dibenzofurans. These findings are in good agreement with the reports of Cornejo et al. [6], Castro et al. [7], Le Pogam [8], Musharraf et al. [9] and Parrot et al. [10].

#### ***Depsides*** 

Seven depsides were identified and assigned to peaks 7, 31, 63, 70–72 and 81 using UHPLC-DAD-MS-MS. Peak 7 and peak 31 were identified as haemathamnolic acid isomer and lecanoric acid whose molecular anions were at *m*/*z* 403.0675 and 317.0670, respectively. Peak 63, 70, 71 and 72 were identified as methyl-8-hydroxy-4-*O*-demethylbarbatate, barbatic acid, sekikaic acid, and 8-hydroxybarbatic acid respectively. Besides, peak 81 was identified as perlatolic acid which showed an [M − H]^−^ ion at *m*/*z* 443.2077 and its fragmentation produced ions at *m*/*z* 223.0973, 205.0867 and 179.1073.

#### ***Depsidones*** 

Twelve depsidones corresponding to peaks 9, 18, 20, 21, 23–25, 29, 32, 35, 39 and 73 were identified (Table 1). Peak 9 was identified as siphulellic acid isomer ([M − H]^−^ ion at *m*/*z* 401.0518) and its fragmentation produced ions at 359.0410 and 240.0427. Peak 18, 20, 21, 23–25, 29, 32, 35, 39 and 73 were identified as salazinic acid, physodalic acid, constictic acid, hypoconstictic acid, connorstictic acid, menegazziaic acid, siphulellic acid, galbinic acid, norstictic acid, cryptostictic acid, and lobaric acid respectively.

#### ***Lipids*** 

Seventeen polyhydroxylated lipids were identified: peaks 34, 37, 41–43, 47, 52, 54, 55, 57, 59, 61, 66, 68, 75 and 84, 85 using UHPLC-ESI-MS-MS analysis.

#### ***Unknown Compounds*** 

Eight compounds were detected in this extract: peaks 2, 3, 5, 11, 13, 14, 17 and peak 27 but none of them was identified.

#### ***Other Compounds*** 

Finally, a dibenzofuran corresponding to usnic acid (peak 78) was detected and identified in this extract.

Among the compounds present in the four-species, we found connorstictic acid (peak 24), tetrahydroxydocosanoic acid (peak 34), 6-ethyl-6-*n*-pentylpentadecan-4,5,7,8,15-pentol-15-acetate (peak 43), tetrahydroxytricosanoic acid (peak 37), methyl-8-hydroxy-4-*O*-demethylbarbatate (peak 63) and usnic acid (peak 78). Furthermore, in all species except *U. antarctic* the following compounds were detected: salazinic acid, lecanoric acid, norstictic acid, and sekikaic acid.

According to Singh et al. 2016 [2] to date more than 60 compounds belonging to different classes such as depsides, depsidones, depsones, lactones, quinones, fatty acids, phenols, polysaccharides and dibenzofurans have been reported from *Usnea* species. In this work we have identified 76 lichen substances using UHPLC-ESI-OT-MS-MS in these four *Usnea* species. Several fatty acids have been previously reported from *Usnea* genus: bourgeanic acid, caperatic acid, murolic acid, isomuronic acid, murotic acid, lichesterinic acid, neuropogolic acid, protolichesteric acid, 18*R*-hydroxydihydroalloprotolichesterenic acid, methyl 3,4-dicarboxy-3-hydroxy-19-oxoeicosanoate, and 2-methylene-3*R*-carboxy-18*R*-hydroxynonadecanoic acid.

We found eleven lipids in *U. barbata*, nine in *U. antarctica*, eighteen in *U. rubicunda* and seventeen in *U. subfloridana* but the following lipids were reported in all mentioned species tetrahydroxydocosanoic acid (peak 34), tetrahydroxytricosanoic acid (peak 37), and 6-ethyl-6-*n*-pentylpentadecan-4,5,7,8,15-pentol-15-acetate (peak 43). Moreover, both in *U. rubicunda* and *U. subfloridana* methyl 3,4-dicarboxy-3-hydroxy-19-oxoeicosanoate (peak 55), neodihydromurolic acid (peak 59), murolic acid (peak 61), muronic acid (peak 66), norcaperatic acid (peak 75), and caperatic acid (peak 84) were identified, following the same trend as reported in the *Usnea* genus. The other lipids mentioned here are reported for the first time in these species and in the genus.

So far, twenty-three depsides have been reported from *Usnea* genus according to Huneck et al. 1996 [3] and Singh et al. 2016 [2]. In this study, we found twenty depsides distributed as: thirteen in *U. barbata*, five in *U. antarctica*, nine in *U. rubicunda* and seven in *U. subfloridana*. We identified barbatolic acid (peak 8), thamnolic acid (peak 10), squamatic acid (peak 15), baeomycesic acid (peak 58), diffractaic acid (peak 60), barbatic acid (peak 70), and atranorin (peak 79), which were previously reported in the genus *Usnea*. The others depsides identified by us are reported for the first time in the genus.

Regarding depsidones, thirteen compounds have been reported in the genus *Usnea* [2,3], while in this study we could identify seventeen. Among the depsidones reported in *Usnea* genus and identified by us were: protocetraric acid (peak 16), salazinic acid (peak 18), constictic acid (peak 21), menegazziaic acid (peak 25), galbinic acid (peak 32), norstictic acid (peak 35), fumarprotocetraric acid (peak 36), hypoconstictic acid (40), stictic acid (peak 50), and lobaric acid (peak 73). The other depsidones identified in this study are reported for the first time in the genus.

Some eight dibenzofurans have been published in the *Usnea* genus [2,3] so far, but we identified two: placodiolic acid (peak 64) and usnic acid (peak 78). It is necessary to mention that usnic acid has been identified in all the species studied. Thirteen compounds have not been identified, which could be new according to our data. Therefore, these compounds should be worthy of further research (isolation and NMR identification).

A pioneering work to determine the components in crude extracts of lichens using MS/MS was done by Leuckert and Holzmann [11]. At that study its authors identified usnic acid, diffractaic acid, gyrophoric acid, lecanoric acid, orsellinic acid, ovoic acid, thamnolic acid, hypothamnolic acid, divaricatic acid, fumarprotocetraric acid, protocetraric acid, homosekikaic acid and sekikaic acid by their specific fragmentation patterns, without an isolation methodology, in the following lichens: *Alectoria ochroleuca*, *Umbilicaria torrefacta*, *Thamnolia vermicularis*, *Ophioparma ventosa*, *Cladonia cryptochlorophaea* and *Cladonia rei*. The Tomasi group [10] has reported a chemical study of eight chemotypes of *Ramalina siliquosa* using LC-ESI-MS/MS and identified ten compounds—conhypoprotocetraric acid, salazinic acid, peristictic acid, cryptostictic acid, protocetraric acid, stictic acid, norstictic acid, hypoprotocetraric acid, 4-*O*-demethylbarbatic acid, usnic acid—and twenty-two more, which were detected but not identified. In another study, nine compounds were identified using a HPLC-MS/MS approach in nine lichens belonging to the genus *Lichina*, *Collema* and *Roccella* [12]. Among them β-orcinol, orsenillic acid, choline sulphate, roccellic acid, montagnetol, lecanoric acid, erythrin, lepraric acid and acetylportentol were identified based on their fragmentation pathways. Choudhary et al. 2015 [9] studied the lichens *Parmotrema grayana* and *Heterodermia obscurata* using HPLC-ESI-QqTOF-MS/MS on negative ion mode. A total of fifteen compounds were detected and identified from the dichloromethane and methanolic extracts. Finally, Le Pogam et al. [8] proposed the rapid identification of lichen extracts using laser desorption/ionization time of flight mass spectrometry instead of electrospray ionization. The analyzed samples were *Diploicia canescens*, *Evernia prunastri*, *Ophioparma ventosa*, *Pseudevernia furfuracea*, *Roccella fuciformis*, *Xanthoria parietina*, Cladonia portentosa, *flavocetraria nivalis*, *Lecidella asema*, *Ramalina siliquosa*, *Vulpicida pinastri* and *Usnea filipendula* and, in general in each studied species 2–5 compounds were detected. For example in the lichen *Usnea filipendula* only salazinic acid and usnic acid were detected. 

Regarding the biological activity of Usnea species, several methanolic extracts of *U. filipendula*, *U. antarctica*, *U. barbata*, *U. complanta*, *U. longissima*, *U. lapponica*, *U. ghattensis*, *U. fasciata*, *U. rubicunda*, *U. siamensis*, and *U. articulate* have been evaluated for anticancer, antioxidant, antiviral, antibacterial, antimycobacterial, anti-inflammatory, cytotoxicity, antigenotoxic, antitumoral, antifungal, antiulcer, antiplatelet, antithrombotic, hepatoprotective and melanogenesis inhibitory activity [2].

**Figure 5:**
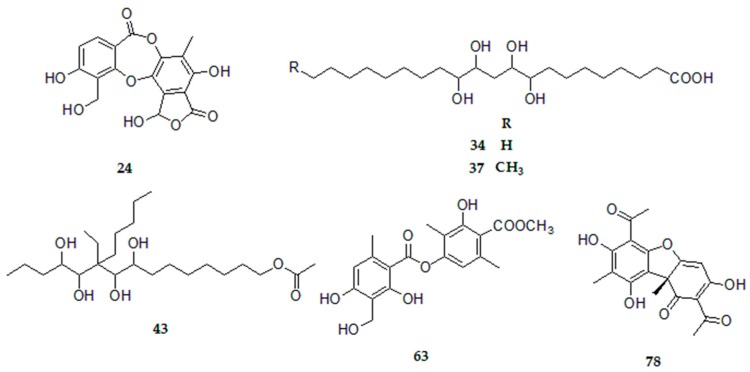
Chemical structures of similar compounds in *U. antarctica*, *U. barbata*, *U. subfloridana* and *U. rubicunda*.

In the study conducted in the four species of the genus *Usnea* eighty six compounds were identified in total, and of these only six compounds (connorstictic acid (24), tetrahydroxydocosanoic acid (34), tetrahydroxytricosanoic acid (37), 6-ethyl-6-*n*-pentylpentadecan-4,5,7,8,15-pentol-15-acetate (43), methyl 8-hydroxy-4-*O*-demethylbarbatate (63) and usnic acid (78), Figure 5) were reported previously in the four species;. The species *U. barbata*, *U. subfloridana* and *U. rubicunda* showed nearly 45 metabolites distributed among the lipid, depsidone, depside, diphenylether, dibenzofuran and chromone classes, while *U. barbata* was the only species to show the presence of β-alectoronic acid (62). The species *U. antarctica* from the Antarctic continent showed the presence of only 21 compounds distributed in six families of secondary metabolites arranged as aromatic, lipid, depsidone, depside, dibenzofuran and chromone classes and unlike the other species studied in this lichen we could detect aromatic compounds.

## 3. Materials and Methods

### 3.1. Lichen Material

The lichen specimen *Usnea barbata* (30 g) was collected at “Longavi”, VII Region, Talca, Chile, in 2015, while *U. antarctica* (50 g) was collected in Ardley Island, Chilean Antarctic, in 2014. Voucher specimens number: UB-19092015 and UA-01032014 were deposited in the Extreme Natural Product Laboratory. Prof. Dr. Reinaldo Vargas confirmed their identity. The species *U. rubicunda* (21 g) and *U. subfloridana* (20 g) were collected in “Combeima river basin”, Ibagué-Tolima, Colombia by Prof. O. Garcia and Prof. A. Torres-Benítez, voucher specimens (COL-015 and COL-016) were deposited in the herbarium of Universidad Distrital Francisco José de Caldas and Prof. Alejandra Suárez Corredor confirmed their identity.

### 3.2. UHPLC-Orbitrap-ESI-MS-MS

#### 3.2.1. Sample Preparation

About 3 g of each lichen species were macerated with methanol (three times, 30 mL each time, 3 days/extraction). The solutions were concentrated to obtain 10 mg (*U. barbata*), 8 mg (*U. antarctica*), 18 mg (*U. rubicunda*) and 22 mg (*U. subfloridana*) of extracts, respectively.

#### 3.2.2. Instruments

A Thermo Scientific Dionex Ultimate 3000 UHPLC system equipped with a quaternary Series RS pump and a Thermo Scientific Dionex Ultimate 3000 Series TCC-3000RS column compartments with a Thermo Fisher Scientific Ultimate 3000 Series WPS-3000RS autosampler and a rapid separations PDA detector controlled by Chromeleon 7.2 Software (Thermo Fisher Scientific, Waltham, MA, USA and Dionex Softron GmbH, a part of Thermo Fisher Scientific, Bremen, Germany) hyphenated with a Thermo high resolution Q Exactive focus mass spectrometer (Thermo, Bremen, Germany) were used for analysis. The chromatographic system was coupled with the MS with a Heated Electrospray Ionization Source II (HESI II). Nitrogen (purity > 99.999%) obtained from a Genius NM32LA nitrogen generator (Peak Scientific, Billerica, MA, USA) was employed as both the collision and damping gas. Mass calibration for the Orbitrap™ was performed once a week, in both negative and positive modes, to ensure a working mass accuracy lower than or equal to 5 ppm. Cafeine and *n*-butylamine (Sigma Aldrich, St. Louis, MO, USA) were the calibration standards for positive ions and buspirone hydrochloride, sodium dodecyl sulfate, and taurocholic acid sodium salt (Sigma Aldrich) were used to calibrate the mass spectrometer. These compounds were dissolved in a mixture of acetic acid, acetonitrile, water and methanol (Merck, Darmstadt, Germany) and were infused using a Chemyx Fusion 100 syringe pump (Thermo Fisher Scientific, Bremen, Germany). XCalibur 2.3 software (Thermo Fisher Scientific, Bremen, Germany) and Trace Finder 3.2 (Thermo Fisher Scientific, San José, CA, USA) were used for UHPLC control and data processing, respectively. Q Exactive 2.0 SP 2 from Thermo Fisher Scientific was used to control the mass spectrometer.

#### 3.2.3. LC Parameters

An UHPLC C18 column (Acclaim, 150 mm × 4.6 mm ID, 5 m, Thermo Fisher Scientific, Bremen, Germany) operated at 25 °C was employed. The detection wavelengths were 254, 280, 320 and 440 nm. PDA was recorded from 200 to 800 nm, and mobile phases were 1% formic aqueous solution (A) and acetonitrile (B). The gradient program (time (min), % B) was: (0.00, 5); (5.00, 5); (10.00, 30); (15.00, 30); (20.00, 70); (25.00, 70); (35.00, 5) and 12 min for column equilibration before each injection. The flow rate was 1.00 mL min^−1^, and the injection volume was 10 μL. Standards and lichen extracts dissolved in methanol were kept at 10 °C inside the autosampler.

#### 3.2.4. MS Parameters

The HESI parameters were as follows: sheath gas flow rate 75 units; aux. gas unit flow rate 20; capillary temperature 400 °C; aux gas heater temperature 500 °C; spray voltage 2500 V (for ESI−); and S lens RF level 30. Full scan data in positive and negative was acquired at the resolving power of 70,000 FWHM (full width half maximum) at *m*/*z* 200. For the compounds of interest, a scan range of *m*/*z* 100–1000 was chosen; the automatic gain control (AGC) set at 3 × 10^6^ and the injection time set to 200 ms. Scan-rate was set at 2 scans s^−1^. External calibration was performed using a calibration solution in positive and negative modes. For confirmation purposes, a targeted MS/MS analysis was performed using the mass inclusion list, with a 30 s time window, with the Orbitrap spectrometer operating both in positive and negative mode at 17,500 FWHM (*m*/*z* 200). The AGC target was set to 2 × 10^5^, with the maximun injection time of 20 ms. The precursor ions are filtered by the quadrupole which operates at an isolation window of *m*/*z* 2. The fore vacuum, high vacuum and ultrahigh vacuum were maintained at approximately 2 mbar, from 105 and below 1010 mbar, respectively. Collision energy (HCD cell) was operated at 30 kv. Detection was based on calculated exact mass and on retention time of target compounds, as shown in Table 1. The mass tolerance window was set to 5 ppm for the two modes.

## 4. Conclusions

In the present study, the use of hyphenated UHPLC-ESI-OT-MS-MS applied to *Usnea* methanolic extracts resulted in the identification of 73 compounds for the first time. The study indicates that lipids, depsides, depsidones, and dibenzofurans were the main compounds detected. This report represents a contribution to the better understanding of the phytochemistry of *Usnea* species.

## Figures and Tables

**Table 1:** Identification of metabolites in *Usnea* species by UHPLC-ESI-MS-MS.

Peak	Tentative Identification	[M − H]^−^	Retention Time (min)	Theoretical Mass (*m*/*z*)	Measured Mass (*m*/*z*)	Accuracy (ppm)	Metabolite Type	MS^2^ Ions (ppm)	Lichens
1	Unknown	C_21_H_19_O_12_	11.09	463.0882	463.0883	0.4	-	-	UB
2	Unknown	C_22_H_19_O_10_N_2_	11.21	471.1040	471.1045	1.1	-	-	UB, US, UR
3	Unknown	C_18_H_13_O_10_	11.42	389.0514	389.0515	0.2	-	-	UB, US, UR
4	Unknown	C_24_H_25_O_15_	11.74	553.1193	553.1195	0.4	-	-	UB
5	Unknown	C_22_H_18_O_11_N	11.80	472.0880	472.0886	1.3	-	-	UB, US, UR
6	Conprotocetraric acid	C_18_H_15_O_9_	12.27	375.0722	375.0724	0.5	d	357.0618; 313.0722 295.0611; 251.0710	UR
7	Haemathamnolic acid	C_19_H_15_O_10_	12.29	403.0671	403.0675	0.9	d	209.0002	US
8	Barbatolic acid	C_18_H_13_O_10_	12.40	389.0514	389.0516	0.2	d	167.0342; 121.0285	UB
9	Siphulellic acid isomer	C_19_H_13_O_10_	12.62	401.0509	401.0518	0.7	D	359.0410; 240.0427	US, UR
10	Thamnolic acid	C_19_H_15_O_11_	12.64	419.0620	419.0619	0.2	d	375.0725; 167.0344	UB
11	Unknown	C_29_H_21_O_9_	12.88	513.1186	513.1149	7.2	-	-	UB, US
12	Unknown	C_21_H_19_O_11_	13.06	447.0927	447.0935	1.8	-	-	UR
13	Unknown	C_20_H_15_O_11_	13.64	431.0620	431.0620	0.0	-	-	UB, US
14	Unknown	C_21_H_22_O_15_	14.01	514.0959	514.0988	5.6	-	-	UB, US
15	Squamatic acid	C_19_H_17_O_9_	14.17	389.0878	389.0880	0.5	d	211.0260	UR
16	Protocetraric acid	C_18_H_13_O_9_	14.43	373.0565	373.0567	0.5	D	311.0559; 267.0657	UR
17	Unknown	C_22_H_18_O_10_N	14.88	456.0931	456.0936	1.1	-	-	UB, US, UR
18	Salazinic acid	C_18_H_11_O_10_	15.11	387.0358	387.0352	0.2	D	269.0464; 241.0504 151.0394; 123.0445	UB, US, UR
19	Conhypoprotocetraric acid	C_18_H_15_O_8_	15.60	359.0772	359.0775	0.8	d	253.0870	UR
20	Physodalic acid	C_20_H_15_O_10_	15.79	415.0671	415.0674	0.7	D	387.0712; 343.0815	US
21	Constictic acid	C_19_H_13_O_10_	16.32	401.0514	401.0516	0.5	D	357.0618; 313.0718	US, UR
22	Haemathamnolic acid isomer	C_19_H_15_O_10_	17.40	403.0665	403.0672	1.7	d	373.0568	UB
23	Hypoconstictic acid derivative	C_19_H_15_O_9_	17.55	387.0722	387.0724	0.5	D	343.0824; 299.0925	US, UR
24	Connorstictic acid	C_18_H_13_O_9_	18.35	373.0565	373.0568	0.8	D	329.0666; 181.0555	UB, UA, US, UR
25	Menegazziaic acid	C_18_H_13_O_9_	18.53	373.0565	373.0567	0.7	D	329.0667; 167.0344 151.0395	US, UR
26	tetrahydroxyeicosanoic acid	C_20_H_39_O_6_	18.64	375.2752	375.2752	0.0	L	-	UB, UR
27	Unknown	C_21_H_19_O_10_	18.77	431.0978	431.0986	1.8	-	-	US, UR
28	Pentahydroxytricosanoic acid	C_23_H_45_O_7_	19.16	433.3171	433.3174	0.7	L	-	UR
29	Siphulellic acid	C_19_H_13_O_10_	19.27	401.0509	401.0514	1.6	D	253.0505; 149.0238 123.0444	UB, US
30	Tetrahydroxyheneicosanoic	C_21_H_41_O_6_	19.27	389.2909	389.2010	0.3	L	-	UA, UR
31	Lecanoric acid	C_16_H_13_O_7_	19.44	317.0667	317.0661	1.9	d	167.0343; 149.0237 123.0444	UB, US, UR
32	Galbinic acid	C_20_H_13_O_11_	19.66	429.0458	429.0463	1.4	D	269.0456; 149.0238 123.0443	UB, US
33	Pentahydroxytetracosanoic acid	C_24_H_47_O_7_	19.69	447.3327	447.3307	4.5	L	-	UA, UR
34	tetrahydroxydocosanoic acid	C_22_H_43_O_6_	19.80	403.3065	403.3067	0.6	L	-	UB, UA, US, UR
35	Norstictic acid	C_18_H_11_O_9_	20.09	371.0409	371.0403	1.6	D	327.0509 151.0393; 123.0444	UB, US, UR
36	Fumarprotocetraric acid	C_22_H_15_O_12_	20.11	471.0569	471.0547	4.7	D	355.0441; 311.0545 115.0023	UA
37	tetrahydroxytricosanoic acid	C_23_H_45_O_6_	20.33	417.3222	417.3216	0.4	L	-	UB, UA, US, UR
38	tetrahydroxydocosanoic acid	C_22_H_43_O_6_	20.38	403.3065	403.3066	0.6	L	-	UB
39	Cryptostictic acid	C_19_H_15_O_9_	20.44	387.0716	387.0726	2.6	D	343.0826; 311.0566 267.0661	US, UR
40	Hypoconstictic acid	C_19_H_15_O_9_	20.57	387.0716	387.0705	2.8	D	343.0808; 299.0923	UA
41	Tetrahydroxydioxoheneicosanoic acid	C_21_H_37_O_8_	20.58	417.2494	417.2498	0.9	L	-	US
42	Tetrahydroxytricosanoic acid	C_23_H_45_O_6_	20.74	417.3222	417.3225	0.7	L	-	US, UR
43	6-ethyl-6-*n*-pentylpentadecan-4,5,7,8,15-pentol-15-acetate	C_24_H_47_O_6_	20.80	431.3373	431.3379	1.4	L	-	UB, UA, US, UR
44	Unknown	C_18_H_11_O_8_	20.93	355.0459	355.0462	0.8	-	-	UB
45	Unknown	C_29_H_59_O_7_N_9_	20.95	645.4537	645.4937	0.0	-	-	UR
46	α-acetylconstictic acid	C_21_H_15_O_11_	21.05	443.0620	443.0620	0.0	D	383.0418; 343.0425 269.0457	UB
47	Trihydroxytrioxodocosanoic acid	C_22_H_37_O_8_	21.29	429.2494	429.2496	0.4	L	-	US, UR
48	Gyrophoric acid *	C_24_H_19_O_10_	21.32	467.0978	467.0985	1.3	d	317.0667; 167.0345 149.0238; 123.0443	UB, UA
49	Tetrahydroxyhexacosenoic acid	C_26_H_49_O_6_	21.32	457.3535	457.3510	4.4	L	-	UA
50	Stictic acid	C_19_H_13_O_9_	21.54	385.0565	385.0567	0.5	D	357.0617; 313.0720 179.0347	UB, UR
51	Tetrahydroxypentacosanoic acid	C_25_H_49_O_6_	21.59	445.3535	445.3515	4.5	L	-	UA
52	Tetrahydroxydioxotricosanoic acid	C_23_H_41_O_8_	21.73	445.2807	445.2812	1.1	L	-	US
53	4-*O*-methylnorsekikaic acid	C_21_H_23_O_8_	21.85	403.1398	403.1400	0.4	d	209.0416; 193.0803 165.0916	UR
54	Tetrahydroxyhexacosanoic acid	C_26_H_51_O_6_	22.03	459.3691	459.3693	0.4	L	-	UB, UA, US
55	Methyl 3,4-dicarboxy-3-hydroxy-19-oxoeicosanoate	C_23_H_39_O_8_	22.14	443.2650	443.2653	0.4	L	-	US, UR
56	Nonahydroxyoctacosanoic acid	C_28_H_55_O_11_	22.65	567.3650	567.3667	3.0	L	-	UB, UA
57	Trihydroxytrioxotetracosanoic acid	C_24_H_41_O_8_	22.72	457.2807	457.2810	0.6	L	-	US
58	Baeomycesic acid	C_19_H_17_O_8_	22.88	373.0923	373.0915	2.1	d	-	UA
59	Neodihydromurolic acid	C_21_H_37_O_5_	22.94	369.2646	369.2650	1.1	L	-	US, UR
60	Diffractaic acid	C_20_H_21_O_7_	22.98	373.1293	373.1294	0.3	d	297.1149; 181.0499 177.0549; 137.0600	UB
61	Murolic acid	C_21_H_35_O_5_	23.23	367.2490	367.2494	0.4	L	-	US, UR
62	β-Alectoronic acid	C_28_H_31_O_9_	23.43	511.1968	511.1976	1.3	DE	369.1339; 247.0969 163.0396	UB
63	Methyl 8-hydroxy-4-*O*-demethylbarbatate	C_19_H_19_O_8_	23.58	375.1080	375.1085	1.6	d	343.0818; 181.0500	UB, UA, US, UR
64	Placodiolic acid	C_19_H_19_O_8_	23.65	375.1080	375.1069	2.9	DBF	343.0806; 259.0597 231.0648	UA
65	Hydroxyeicosatrienoic acid	C_20_H_33_O_3_	23.65	321.2435	321.2437	0.6	L	-	UR
66	muronic acid	C_21_H_33_O_5_	23.89	365.2333	365.2338	0.6	L	-	US, UR
67	Tetrahydroxytrioxotricosanoic acid	C_23_H_39_O_9_	23.98	459.2594	459.2602	1.7	L	-	UB
68	Trihydroxytrioxopentacosanoic acid	C_25_H_43_O_8_	24.00	471.2963	471.2970	1.5	L	-	US
69	Divaricatic acid	C_21_H_23_O_7_	24.09	387.1449	387.1452	0.7	d	195.0661; 177.0551 151.0759	UB
70	Barbatic acid	C_19_H_19_O_7_	24.20	359.1136	359.1139	0.8	d	181.0503; 163.0394 137.0601	UB, UA, US
71	Sekikaic acid	C_22_H_25_O_8_	24.31	417.1549	417.1556	1.7	d	225.0764; 209.0815 165.0915	UB, US, UR
72	8-hydroxybarbatic acid	C_19_H_19_O_8_	24.53	375.1080	375.1088	2.4	d	195.0663; 181.0506	UB, UA, US
73	Lobaric acid	C_25_H_27_O_8_	24.73	455.1711	455.1712	0.4	D	411.1815; 367.1909 352.1681; 296.1048	UB, UA, US
74	Boninic acid	C_25_H_31_O_8_	24.83	459.2024	459.2027	0.6	d	209.1180	UR
75	Norcaperatic acid	C_20_H_35_O_7_	24.95	387.2388	387.2392	0.8	L	-	US, UR
76	Tetrahydroxytrioxotetracosanoic acid	C_24_H_41_O_9_	25.01	473.2756	473.2756	0.0	L	-	UB
77	Ethyl-4-*O*-methylolivetolcarboxylate	C_15_H_21_O_4_	25.34	265.1445	265.1466	7.9	A	-	UA
78	Usnic acid *	C_18_H_15_O_7_	26.04	343.0818	343.0823	1.5	DBF	328.0591; 259.0609; 231.0661	UB, UA, US, UR
79	Atranorin	C_19_H_17_O_8_	26.31	373.0923	373.0929	1.6	d	177.0187; 163.0394	UB, UR
80	trioxotricosanoic acid	C_23_H_39_O_5_	26.45	395.2803	395.2805	0.5	L	-	UR
81	Perlatolic acid	C_25_H_31_O_7_	26.77	443.2075	443.2077	0.5	d	223.0973; 205.0867 179.1073	US
82	Hexadecadienoic acid	C_16_H_27_O_2_	26.96	251.2017	251.2016	0.1	L	-	UB
83	Dihydroxyheptadecatrienoic acid	C_17_H_27_O_4_	27.31	295.1915	295.1916	0.3	L	-	UB
84	Caperatic acid	C_21_H_37_O_7_	28.10	401.2545	401.2548	0.7	L	-	US, UR
85	Hydroxytrioxotricosanoic acid	C_23_H_39_O_6_	28.87	411.2752	411.2754	0.4	L	-	US, UR
86	Chloroatranorin	C_19_H_16_O_8_Cl	28.96	407.0534	407.0541	1.5	d	228.9906; 210.9800 163.0394	UB

* Identified by spiking experiments with an authentic compound. A = Aromatic; L = Lipid; D = depsidone; d = depside; DE = diphenylether; DBF = dibenzofuran. C = Chromone. UA: *Usnea antarctica*; UB: *Usnea barbata*; UR: *Usnea rubicunda*; US: *Usnea subfloridana*; MS^2^ = Daughter ions.

## Supplementary Materials

The following are available online. Table S1: Structure of the compounds identified by UHPLC-ESI-MS-MS from *Usnea* species.
