# Supplementary material for: Secondary Metabolite Profiling of Species of the Genus Usnea by UHPLC-ESI-OT-MS-MS
Source: Molecules. 2017 Dec 27;23(1):54. doi: 10.3390/molecules23010054 (PMC6017147; doi:10.3390/molecules23010054)
Supplement: Supplementary file 1 [file molecules-23-00054-s001.pdf]

Sopporting data

Secondary metabolites profiling of species of the genus *Usnea* by UHPLC-ESI-OT-MS-MS

Francisco Salgado[1], Laura Albornoz[1], Carmen Cortéz[1], Elena Stashenko[2], Kelly Urrea-Vallejo[3], Edgar Nagles[1], Cesar Galicia-Virviescas[1], Alberto Cornejo[4], Alejandro Ardiles[5], Mario Simirgiotis[6,7], Olimpo García-Beltrán[3], * and Carlos Areche[1,*]

Table S1. Structure of the compounds identified by UHPLC-ESI-OT-MS-MS from *Usnea* species

Peak	Name	Structure
6	Conprotocetraric acid	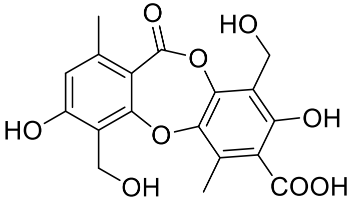
7	Haemathamnolic acid	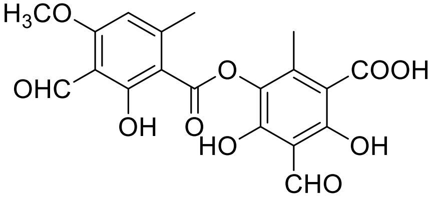
8	Barbatolic acid	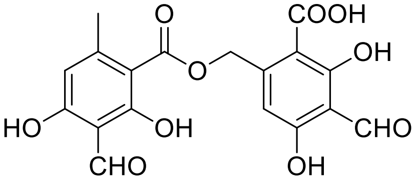
9	Siphulellic acid isomer	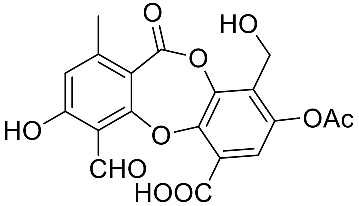

10	Thamnolic acid	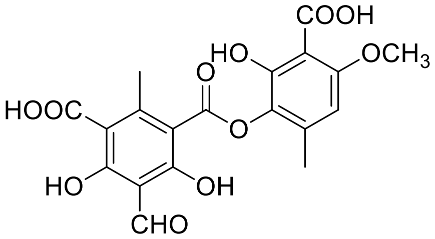
15	Squamatic acid	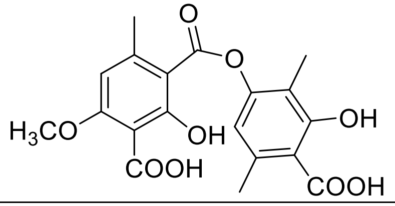
16	Protocetraric acid	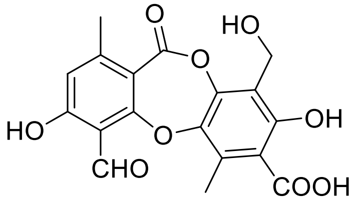
18	Salazinic acid	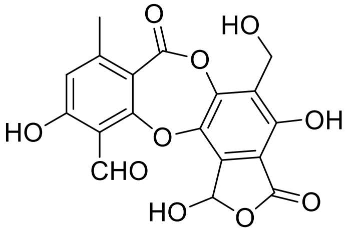
19	Conhypoprotocetraric acid	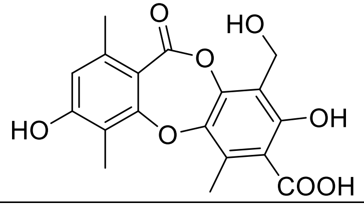

20	Physodalic acid	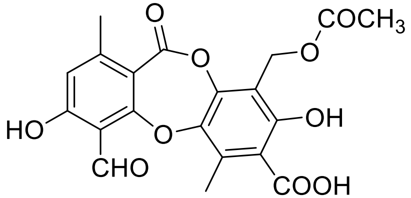
21	Constictic acid	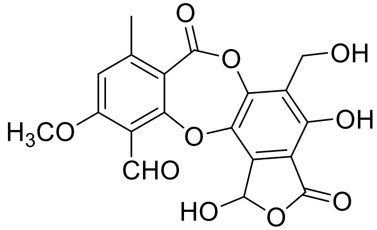
22	Haemathamnolic acid isomer	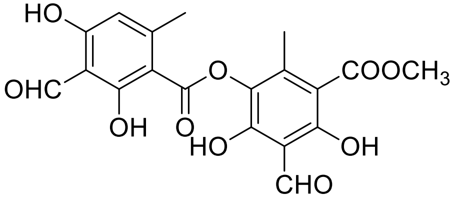
23	Hypoconstictic acid derivative	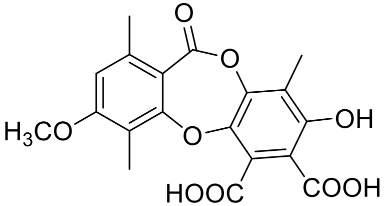
24	Connorstictic acid	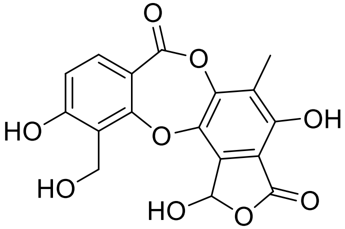

25	Menegazziaic acid	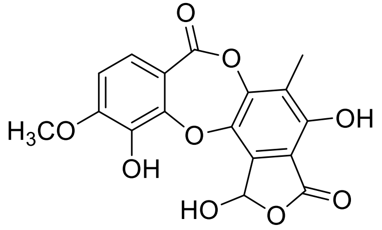
26	tetrahydroxyeicosanoic acid	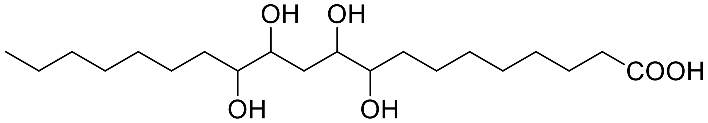
28	Pentahydroxytricosanoic acid	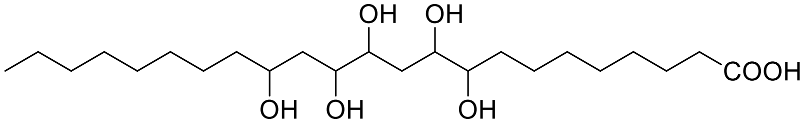
29	Siphulellic acid	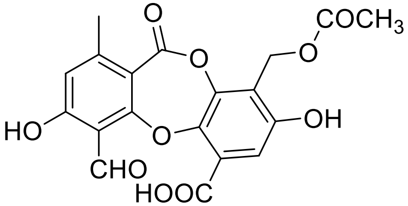
30	Tetrahydroxyheneicosanoic	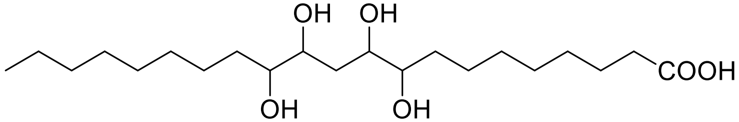
31	Lecanoric acid	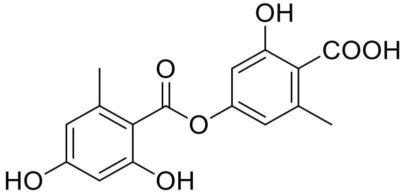

32	Galbinic acid	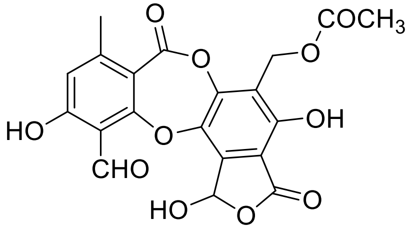
33	Pentahydroxytetracosanoic acid	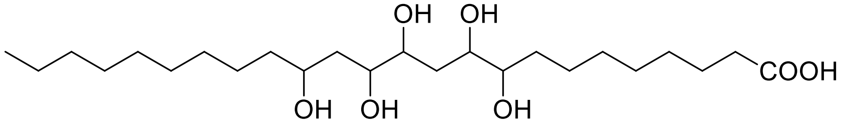
34	tetrahydroxydocosanoic acid	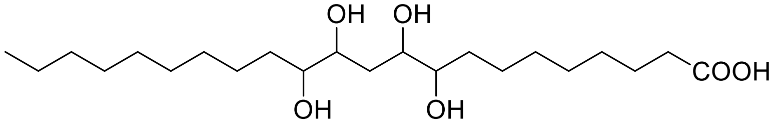
35	Norstictic acid	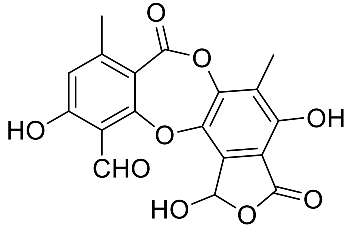
36	Fumarprotocetraric acid	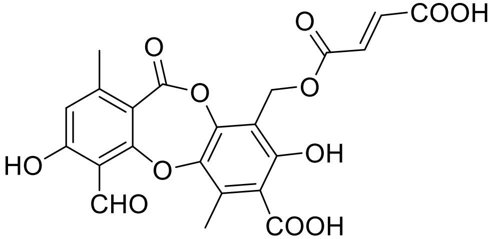
37	tetrahydroxytricosanoic acid	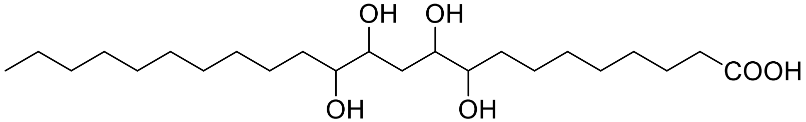

38	tetrahydroxydocosanoic acid	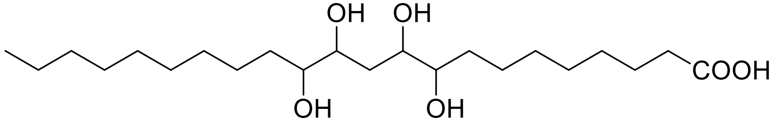
39	Cryptostictic acid	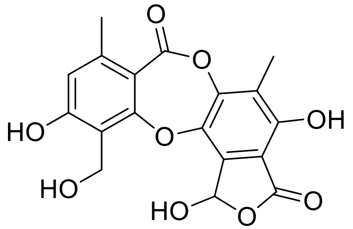
40	Hypoconstictic acid	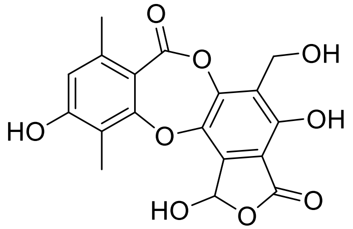
41	Tetrahydroxydioxoheneicosanoic acid	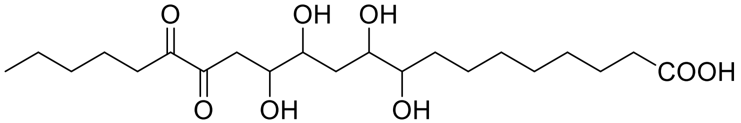
42	Tetrahydroxytricosanoic acid	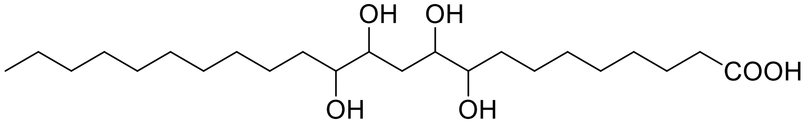
43	6-ethyl-6-n-pentylpentadecan-4,5,7,8,15-pentol-15-acetate	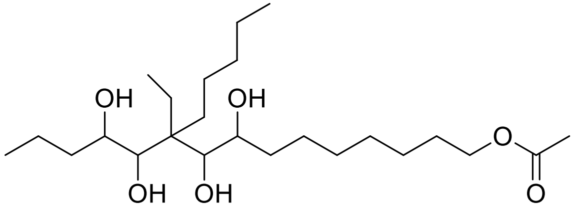

46	α-acetylconstictic acid	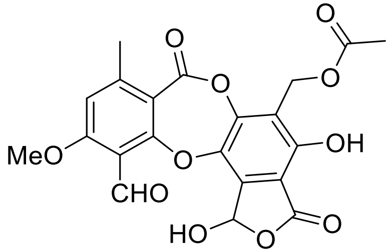
47	Trihydroxytrioxodocosanoic acid	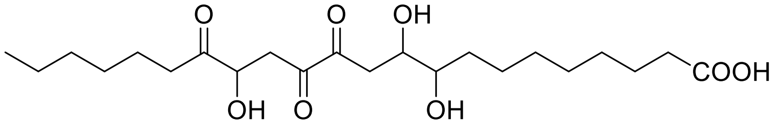
48	Gyrophoric acid*	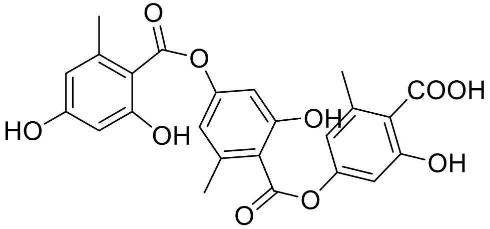
49	Tetrahydroxyhexacosenoic acid	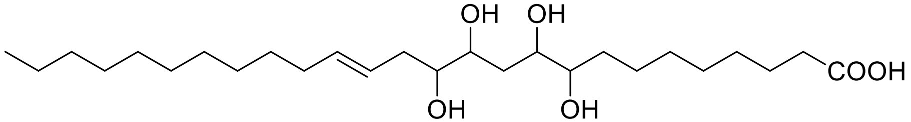
50	Stictic acid	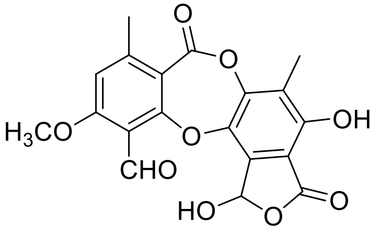

51	Tetrahydroxypentacosanoic acid	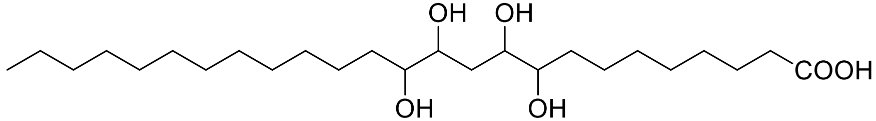
52	Tetrahydroxydioxotricosanoic acid	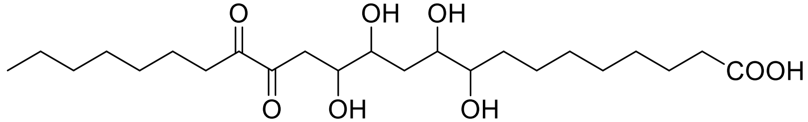
53	4-O-methylnorsekikaic acid	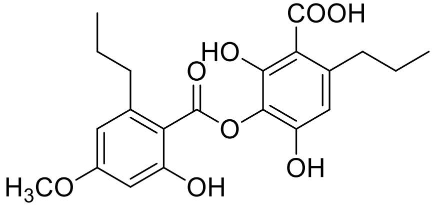
54	Tetrahydroxyhexacosanoic acid	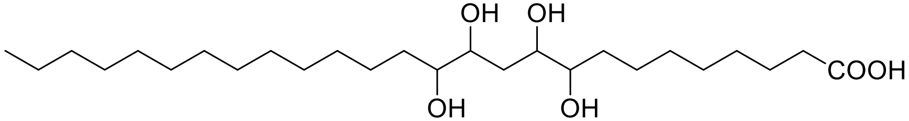
55	Methyl 3,4-dicarboxy-3-hydroxy-19-oxoeicosanoate	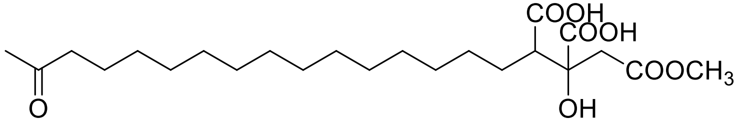
56	Nonahydroxyoctacosanoic acid	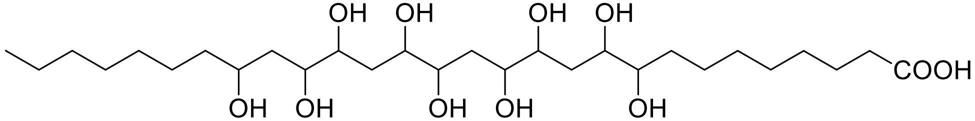
57	Trihydroxytrioxotetracosanoic acid	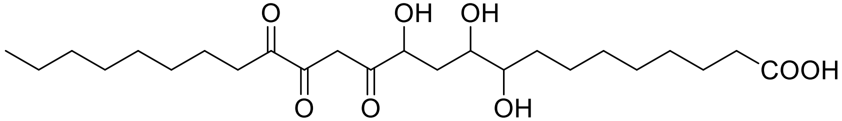

58	Baeomycesic acid	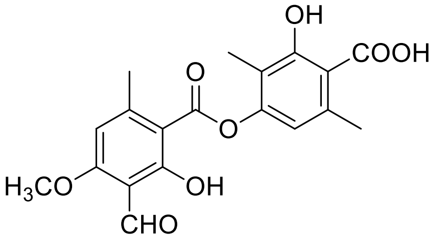
59	Neodihydromurolic acid	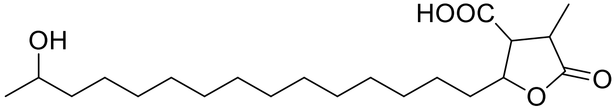
60	Diffractaic acid	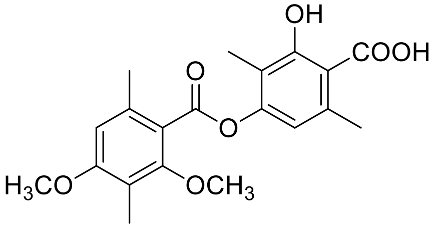
61	Murolic acid	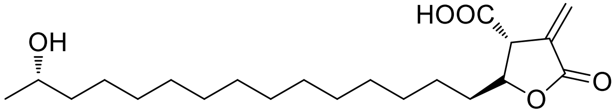

62	β-Alectoronic acid	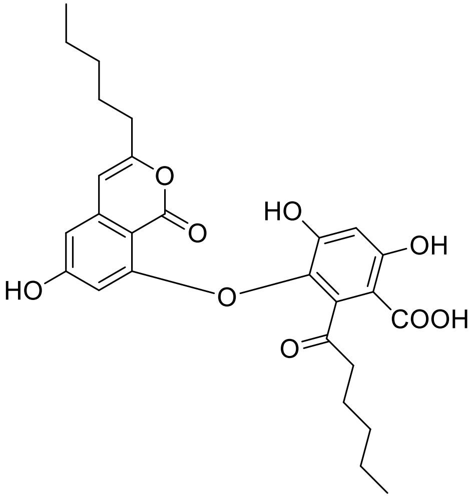
63	Methyl 8-hydroxy-4-O-demethylbarbatate	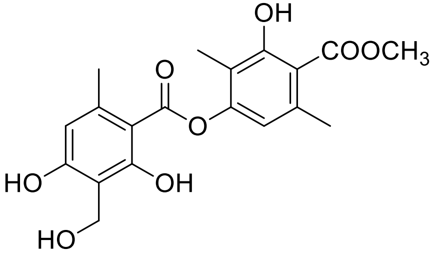
64	Placodiolic acid	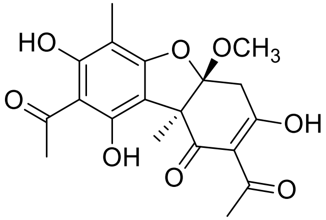
65	Hydroxyeicosatrienoic acid	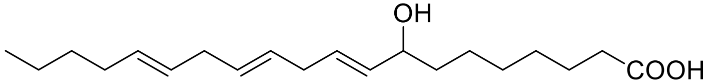

66	Muronic acid	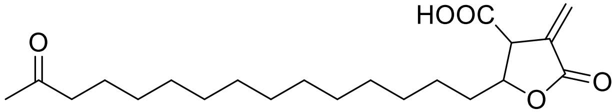
67	Tetrahydroxytrioxotricosanoic acid	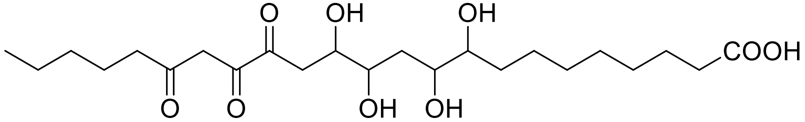
68	Trihydroxytrioxopentacosanoic acid	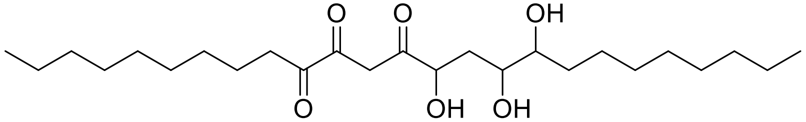
69	Divaricatic acid	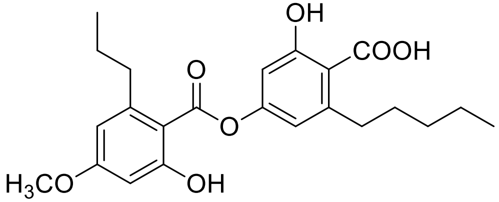
70	Barbatic acid	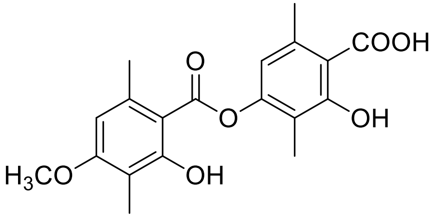
71	Sekikaic acid	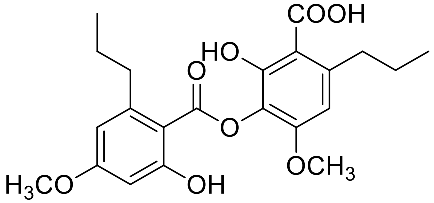

72	8-hydroxybarbatic acid	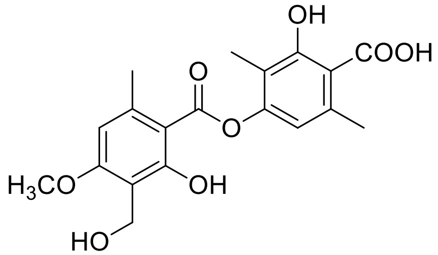
73	Lobaric acid	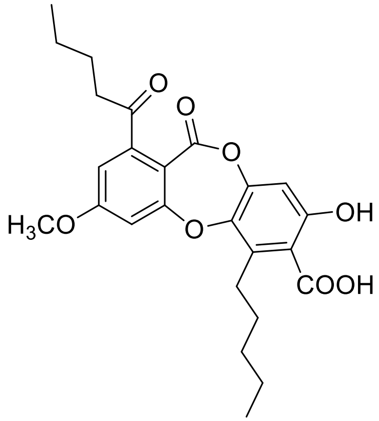
74	Boninic acid	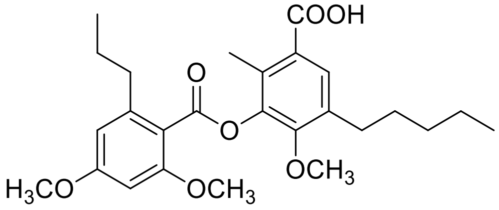
75	Norcaperatic acid	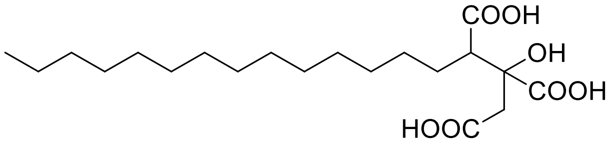

76	Tetrahydroxytrioxotetracosanoic acid	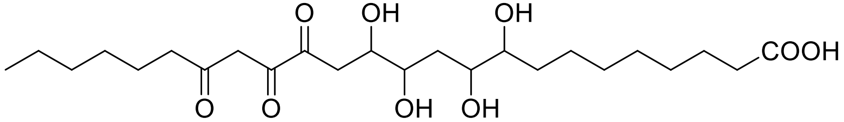
77	Ethyl-4-O-methylolivetolcarboxylate	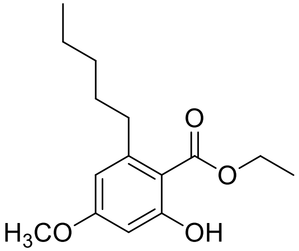
78	Usnic acid*	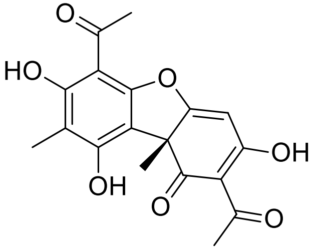
79	Atranorin	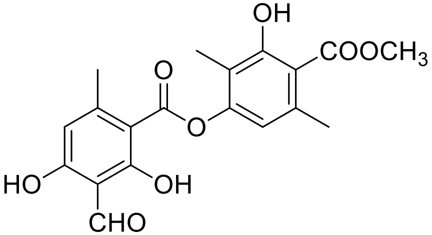
80	Trioxotricosanoic acid	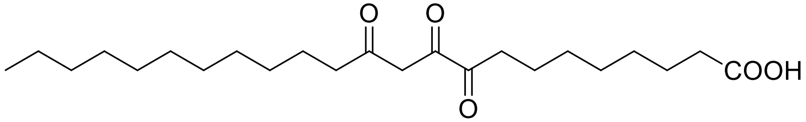

81	Perlatolic acid	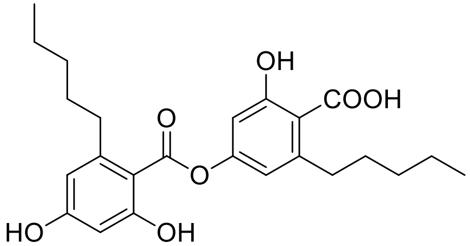
82	Hexadecadienoic acid	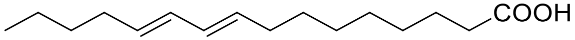
83	Dihydroxyheptadecatrienoic acid	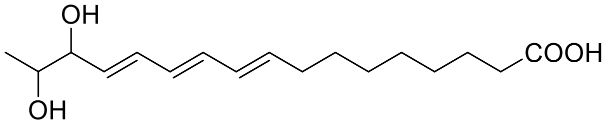
84	Caperatic acid	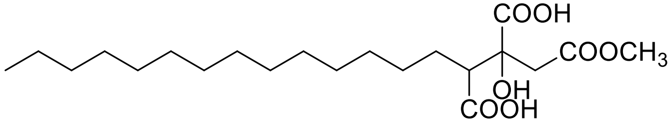
85	Hydroxytrioxotricosanoic acid	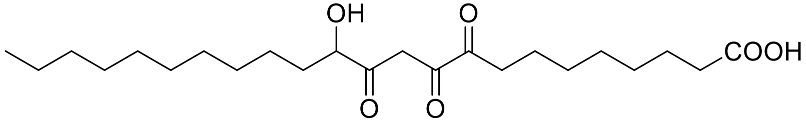
86	Chloroatranorin	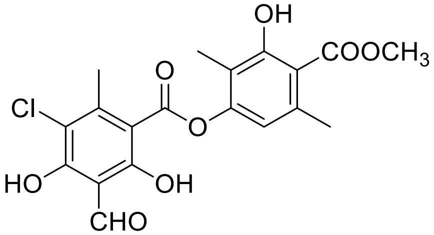
